# Supplementary material for: Heat adaptation of phage T7 under an extended genetic code
Source: Virus Evol. 2021 Dec 1;7(2):veab100. doi: 10.1093/ve/veab100 (PMC8923235; doi:10.1093/ve/veab100)
Supplement: veab100_Supp [file veab100_supp.zip › Sequencing_Analysis.zip!/Sequencing_Analysis/S2/evidence/SNP_12.html]

BRESEQ :: Evidence


| Predicted mutation | | | | | | |
| --- | --- | --- | --- | --- | --- | --- |
| evidence | seq id | position | mutation | annotation | gene | description |
| RA | NC\_001604.1 | 29,486 | G→A | intergenic (–/–) | – / – | –/– |

| Read alignment evidence... | | | | | | | | | | | |
| --- | --- | --- | --- | --- | --- | --- | --- | --- | --- | --- | --- |
|  | seq id | position | | ref | new | freq | score (cons/poly) | reads | annotation | genes | product |
| \* | NC\_001604.1 | 29,486 | 0 | G | A | 99.8% | 14063.0 / NA | 5342 | intergenic (–/–) | *–/–* | –/– |
| Reads supporting (aligned to +/- strand):  **ref** base G (5/2);  **major** base A (2591/2728);  **minor** base C (9/3);  **total** (2607/2735) | | | | | | | | | | | |
| Fisher's exact test for biased strand distribution *p*-value = 8.51e-02 | | | | | | | | | | | |
| Kolmogorov-Smirnov test that lower quality scores support variant *p*-value = 1.00e+00 | | | | | | | | | | | |

Only 100 of 5369 total aligned reads displayed.  

`CGCTCTCATGGTCACAGCACAACGTTCTCAGTTTGAGACTGACGCGAAGCTGAACGAGCAGTATCGCTTGAAGATTAACTCTGCGCTGAACCAAGAGGACCCAAGGACAGCTTGGGAGATGCTTCAAGGTATCAAGGCTGAACTAGATAAGGTCCAACCTGATGAGCAGATGACACCACAACGTGAGTGGCTAATCTCCGCACAGGAACAAGTTCAGAATCAGATGAACGCATGGACGAAAGCTCAGGCCAAGGCTC‑TGGACGATTCCATGAAGTCAATGAACAAACTTGACGTAATCGACAAGCAATTCCAGAAGCGAATCAACGGTGAGTGGGTCTCAACGGATTTTAAGGATATGCCAGTCAACGAGAACACTGGTGAGTTCAAGCATAGCGATATGGTTAACTACGCCAATAAGAAGCTCGCTGAGATTGACAGTATGGACATTCCAGACGGTGCCAAGGATGCTATGAAGTTGAAGTACCTTCAAGCGGACTCTAAGGACGGAGCATTCCGTACAGCCATCGGAACCATGGTCACTGACGCTGGTCAAGAGTGGTCTGCCGCTGTGATTAACGGTAAGTTACCAGAACGAAC  >  NC_001604.1/29188‑29784`  

                                                                                                                                                                                                                                                                                                           `|`                                                                                                                                                                                                                                                                                                            
  `ctctCATGGTCACAGCACAACGTTCTCAGTTTGAGACTGACGCGAAGCTGAACGAGCAGTATCGCTTGGAGATTAACTCTGCGCTGAACCAAGAGGACCCAAGGACAGCTTGGGCGATGCTTCAAGGTATCAAGGCTGAACTAGATAAGGTCCAACCTGATGAGCAGATGACACCACAACGTGAGTGGCTAATCTCCGCACAGGAACAAGTTCAGAATCTGATGAACGCATGGACGAAAGCTCAGGCCAAGGCTC‑TGGACGATTCCATGAAGTCAATGCACAAACTTGACGTAATcaacaa                                                                                                                                                                                                                                                                                                        >  1:672082/1‑301 (MQ=255)`  
      `cATGGTCACAGCACAACGTTCTCAGTTTGAGACTGACGCGAAGCTGAACGAGCAGTATCGCTTGGAGATTAACTCTGCGCTGAACCAAGAGGACCCAAGGACAGCTTGGGCGATGCTTCAAGGTATCAAGGCTGAACTAGATAAGGTCCAACCTGATGAGCAGATGACACCACAACGTGAGTGGCTAATCTCCGCACAGGAACAAGTTCAGAATCAGATGAACGCATGGACGAAAGCTCAGGCCAAGGCTC‑TGGACGATTCCATGAAGTCAATGAACAAACTTGACGTAATCAACAAGCaa                                                                                                                                                                                                                                                                                                    >  1:379672/1‑301 (MQ=255)`  
          `gTCACAGCACAACGTTCTCAGTTTGAGACTGACGCGAAGCTGAACGAGCAGTATCGCTTGGAGATTAACTCTGCGCTGAACCAAGAGGACCCAAGGACAGCTTGGGGGATGCTTCAGGGTATCAAGGCTGAACTAGATAAGGTCCAACCTGATGAGCAGATGCAACCACAACGTGAGTGGCTCATCTCCGCACAGTAACAAGTTCAGAATCAGATGAACGCATGGACGATAGCACAGGCCACGCCGC‑TGGACGATTCCATGAGGTGAATGAACAAAATTGACATAATCAACAAGCAATTcc                                                                                                                                                                                                                                                                                                >  1:326968/1‑301 (MQ=255)`  
            `cacaGCACAACGTTCTCAGGTTGAGACTGACGCGAAGCTGAACGAGCAGTATAGCTTGGAGATTAACTCTGCGCTGAACCAAGAGGACCCAAGGACAGCTTGGGAGATGCTTCAAGGTATCAAGGCTGAACTAGATAAGGTCCAACCTGATGAGCAGATGACACCACAACGTGAGTGGCTAATCTCCGCACAGGAACAAGTTCAGAATCAGATGAACGCATGGACGAAAGCTCAGGCCAAGGCTC‑TGGACGATTCCATGAAGTCAATGAACAAACTTGACGTAATCAACAAGCAATTCCAg                                                                                                                                                                                                                                                                                              <  1:61260/301‑1 (MQ=255)`  
                 `gggctcggagatgtgtataagagacagcgAAGCTGAACGAGCAGTATCGCTTGGAGATTAACTCTGCGCTGAACCAAGAGGACCCAAGGACAGCTTGGGCGATGCTTCAAGGTATCAAGGCTGAACTAGATAAGGTCCAACCTGATGAGCAGATGACACCACAACGTGAGTGGCTAATCTCCGCACAGGAACAAGTTCAGAATCAGATGAACGCATGGACGAAAGCTCAGGCCAAGGCTC‑TGGACGATTCCATGAAGTCAATGAACAAACTTGACGTAATCAACAAGCAATTCCAGAAGCg                                                                                                                                                                                                                                                                                         <  1:240772/275‑1 (MQ=255)`  
                      `cGTTCTCAGTTTGAGACTGACGCGAAGCTGAACGAGCAGTATCGCTTGGAGATTAACTCTGCGCTGAACCAAGAGGACCCAAGGACAGCTTGGGCGATGCTTCAAGGTATCAAGGCTGAACTAGATAAGGTCCAACCTGATGAGCAGATGACACCACAACGTGAGTGGCTAATCTCCGCACAGGAACAAGTTCAGAATCAGATGAACGCATGGACGAAAGCTCAGGCCAAGGCTC‑TGGACGATTCCATGAAGTCAATGAACAAACTTGACGTAATCAACAAGCAATTCCAGAAGCGAAACa                                                                                                                                                                                                                                                                                    >  1:16490/1‑301 (MQ=255)`  
                              `gTTTGAGACTGACGCGAAGCTGAACGAGCAGTATCGCTTGGAGATTAACTCTGCGCTGAACCAAGAGGACCAAAGGACAGCTTGGGCGATGCTTCAAGGTATCAAGGCTGAACTAGATAAGGTCCAACCTGATGAGCAGATGACACCACAACGTGAGTGGCTAATCTCCGCACAGGAACAAGTTCAGAATCAGATGAACGCATGGACGAAAGCTCAGGCCAAGGCTC‑TGGACGATTCCATGAAGTCAATGAACAAACTTGACGTAATCAACAAGCAATTCCAGAAGCGAATCAACGGTGAg                                                                                                                                                                                                                                                                            <  1:52410/301‑1 (MQ=255)`  
                                     `acagaagcgAAGATGAACGAGCAGTATAGATTGGAGATAAAATCTGCGCTGAACCAAGAGGACCCAAGGACAGCTTGGGCGATGCTTAAAGGTATCAAGGCTGAACTAGATAAGGTCCAACCTGATGAGCAGATGACACCACAACGTGAGTGGCTAATCTCCGCACAGGAACAAGTTCAGAATCAGATGAACGCATGGACGAAAGCTCAGGCCAAGGCTC‑TGGACGATTCCATGAAGTCAATGAACAAACTTGACGTAATCAACAAGCAATTCCAGAAGCGAATCAACGGTGAGTGGGtct                                                                                                                                                                                                                                                                     <  1:532868/295‑1 (MQ=255)`  
                                                   `gtataagagacagcGCTTGGAGATTAACTCTGCGCTGAACCAAGAGGACCCAAGGACAGCTTGGGCGATGCTTCAAGGTATCAAGGCTGAACTAGATAAGGTCCAACCTGATGAGCAGATGACACCACAACGTGAGTGGCTAATCTCCGCACAGGAACAAGTTCAGAATCAGATGAACGCATGGACGAAAGCTCAGGCCAAGGCTC‑TGGACGATTCCATGAAGTCAATGAACAAACTTGACGTAATCAACAAGCAATTCCAGAAGCGAATCAACGGTGAGTGGGTCTCAACGGATTTTAAg                                                                                                                                                                                                                                                       <  1:74498/288‑1 (MQ=255)`  
                                                   `gatagtacactATCGCCTGGAGCTTAACGCTCCGCTGGCCCACGAGCACCCAAGTCAAGCCTTGGCCATGCTTCAAGGTATGAAAGCTGTACTAGATAAGGTCCATCCGGATGAGCAGACTACACCACAACGTGGGTGGCTGATCTCCGTACAGGAGCAAGTTCATAATCAGAGGAACGCATGGACGAAGGCTCAAGCCAAGGCTC‑TGGACGATTCCATGAAGTCAATGAACATACTTGACGTAATAAACAAGCAATTCCAGAAGCGAATCAACGGTGAGTGGGTCTCAACGCATTTTAAg                                                                                                                                                                                                                                                       <  1:591158/291‑1 (MQ=255)`  
                                                    `aaCGAGCAGTATCGCTTGGAGATTAACTCTGCGCTGAACCAAGAGGACCCAAGGACAGCTTGGGCGATGCTTCAAGGTATCAAGGCTGAACTAGATAAGGTCCAACCTGATGAGCAGATGACACCACAACGTGAGTGGCTAATCTCCGCACAGGAACAAGTTCAGAATCAGATGAACGCATGGACGAAAGCTCAGGCCAAGGCTC‑TGGACGATTCCATGAAGTCAATGAACAAACTTGACGTAATCAACAAGCAATTCCAGAAGCGAATCAACGGTGAGTGGGTCTCAACGGATTTTAAgg                                                                                                                                                                                                                                                      <  1:492803/301‑1 (MQ=255)`  
                                                    `aaCGAGCAGTATCGCTTGGAGATTAACTCTGCGCTGAACCAAGAGGACCCAAGGACAGCTTGGGCGATGCTTCAAGGTATCAAGGCTGAACTAGAAAAGGTCCAACCTGATGAGCAGATGACACAACAACGTGAGTGGCTAATCTCCGCACAGGAACAAGTTCAGAATCAGATGAACGCATGGAAGAAAGCTCAGGCCAAGGCTC‑TGGACGATTCCATGAAGTCAATGAACAAACTTGACGTAATCAACAAGCAATTCCAGAAGCGAATCAACGGTGAGTGGGTCTCAACGGATTTTAAgg                                                                                                                                                                                                                                                      <  1:793007/301‑1 (MQ=255)`  
                                                     `aCGAGCAGTATCGCTTGGAGATTAACTCTGCGCTGAACCAAGAGGACCCAAGGACAGCTTGGGCGATGCTTCAAGGTATCAAGGCTGAACTAGATAAGGTCCAACCTGATGAGCAGATGACACCACAACGTGAGTGGCTAATCTCCGCACAGGAACAAGTTCAGAATCAGATGAACGCATGGACGAAAGCTCAGGCCAAGGCTA‑TGGACGATTCCATGAAGTCAATGAACAAACTTGACGTAATCAACAAGCAATTCCAGAAGCGAATCAACGGTGAGTGGGTCTCAACGGATTTTAAGGa                                                                                                                                                                                                                                                     <  1:75213/301‑1 (MQ=255)`  
                                                       `gAGCAGTATCGCTTGGAGATTAACTCTGCGCTGAACCAAGAGGACCCAAGGACAGCTTGGGCGATGCTTCAAGGTATCAAGGCTGAACTAGATAAGGTACAACCTGATGAGCAGATGACACCACAACGTGAGTGGCTAATCTCCGCACAGGAACAAGTTCAGAATCAGATGAACGCATGGACGAAAGCTCAGGCCAAGGCTC‑TGGACGATTCCATGAAGTCAATGAACAAACTTGACGTAATCAACAAGCAATTCCAGAAGCGAATCAACGGTGAGTGGGTCTCAACGGATTTTAAGGata                                                                                                                                                                                                                                                   >  1:434228/1‑301 (MQ=255)`  
                                                           `ggTATCGCTTGGAGATTAACTCTGCGCTGAACCAAGAGGACCCAAGGACAGCTTGGGCGATGCTTCAAGGTATCAAGGCTGAACTAGATAAGGTCCAACCTGATGAGCAGATGACACCACAACGTGAGTGGCTAATCTCCGCACAGGAACAAGTTCAGAATCAGATGAACGCATGGACGAAAGCTCAGGCCAAGGCTC‑TGGACGATTCCATGAAGTCAATGAACAAACTTGACGTAATCAACAAGCAATTCCAGAAGCGAATCAACGGTGAGTGGGTCTCAACGGATTTTAAGGATATGcc                                                                                                                                                                                                                                               <  1:292156/300‑1 (MQ=255)`  
                                                               `aagagacaggATTAACTCTGCGCTGAACCAAGAGGACCCAAGGACAGCTTGGGCGATGCTTCAAGGTATCAAGGCTTAACTAGATAAGGTCCAACCTGATGAGCAGATGACACCACAACGTGAGTGGCTAATCTCCGCACAGGAACAAGTTCATAATCAGATGAACGCATGGACGAAAGCTCAGGCCAAGGCTC‑TGGACGATTCCATGAAGTCAATGAACAAACTTGACGTAATCAACAAGCAATTCCAGAAGCGAATCAACGGTGAGTGGGTCTCAACGGATTTTAAGGATATGCCAGTc                                                                                                                                                                                                                                           <  1:148410/292‑1 (MQ=255)`  
                                                                             `aCTCTGCGCTGCACCAAGAGGACCCAAGGACAGCTTGGGCGATGCTTCAAGGTATCAAGGCTGAACTAGATAAGGTCCAACCTGATGAGCAGATGACACCACAACGTGAGTGGCTAATCTCCGCACAGGAACAAGTTCAGAATCAGATGAACGCATGGACGAAAGCTCAGGCCAAGGCTC‑TGGACGATTCCATGAAGTCAATGAACAAACTTGACGTAATCAACAAGCAATTCCAGAAGCGAATCAACGGTGAGTGGGTCTCAACGGATTTTAAGGATATGCCAGTCAACGAGAACACTgg                                                                                                                                                                                                                             <  1:202932/301‑1 (MQ=255)`  
                                                                             `aCTCTGCGCTGAACCAATAGACCCCAAGGACAGCTTGGGCGATGCTTAAAGGTATCAAGGCTGAACTAGATAAGGTCAAACCTGATGAGCAGATGACACCACAACGTGAGTGGCTAATCTCCGAACAGGAACAAGTTCAGAATCAGATGAACGCATGGACGAAAGCTCAGGCAAAGGCTA‑TGGACGATTCCATGAAGTCAATGAACAAACTTGACGTAATCAACAAGCAATTCCAGAAGCGAATCAACGGTGAGTGGGTCTCAACGGATTTTAAGGATATGCCAGTCAACGAGAACACTgg                                                                                                                                                                                                                             <  1:372329/301‑1 (MQ=255)`  
                                                                             `aCTCTGCGCTGAACCAAGAGGACCCAAGGACAGCTTGGGCGATGCTTCAAGGTATCAAGGCTGAACTAGATAAGGTCCAACCTGATGAGCAGATGACACCACAACGTGAGTGGCTAATCTCCGCACAGGAACAAGTTCAGAATCAGATGAACGCATGGACGAACGCTCAGGCCAAGGCTC‑TGAACGATTCCATGAAGTCAATGAACAAACTTGACGTAATCAACAAGCAATTCCAGAAGCGAATCAACGGTGAGTGGGTCTCAACGGATTTTAAGGATATGCCAGTCAACGAGAACACTgg                                                                                                                                                                                                                             >  1:804620/1‑301 (MQ=255)`  
                                                                             `aCTCTGCGCTGAACCAAGAGGACCCAAGGACAGCTTGGGCGATGCTTCAAGGTATCAAGGCTGAACTAGATAAGGTCCAACCTGATGAGCAGATGACACCACAACGTGAGTGGCTAATCTCCGCACAGGAACAAGTTCAGAATCAGATGAACGCATGGACGAAAGCTCAGGCCAAGGCTC‑TGGACGATTCCATGAAGTCAATGAACAAACTTGACGTAATCAACAAGCAATTCCAGAAGCGAATCAACGGTGAGTGGGTCTCAACGGATTTTAAGGATATGCCAGTCAACGAGAACACTgg                                                                                                                                                                                                                             >  1:798490/1‑301 (MQ=255)`  
                                                                               `tctGCGCTGAACCAAGAGGACCCAAGGACAGCTTGGGCGATGCTTCAAGGTATCAAGGCTGAACTAGATAAGGTCCAACCTGATGAGCAGATGACACCACAACGTGAGTGGCTAATCTCCGCACAGGAACAAGTTCAGAATCAGATGAACGCATGGACGAAAGCTCAGGCCAAGGCTC‑TGGACGATTCCATGAAGTCAATGAACAAACTTGACGTAATCAACAAGCAATTCCAGAAGCGAATCAACGGTGAGTGGGTCTCAACGGATTTTAAGGATATGCCAGTCAACGAGAACACTGGTg                                                                                                                                                                                                                           <  1:777464/301‑1 (MQ=255)`  
                                                                                  `ataagagacagagagGAACCAAGGACAGCTTGGGCGATGCTTCAAGGTATCAAGGCTGAACTAGATAAGGTCCAACCTGATGAGCAGATGACACCACAACGTGAGTGGCTAATCTCCGCACAGGAACAAGTTCAGAATCAGATGAACGCATGGACGAAAGCTCAGGCCAAGGCTC‑TGGACGATTCCATGAAGTCAATGAACAAACTTGACGTAATCAACAAGCAATTCCAGAAGCGAATCAACGGTGAGTGGGTCTCAACGGATTTTAAGGATATGCCAGTCAACGAGAACACTGGTGAGt                                                                                                                                                                                                                        <  1:46398/290‑1 (MQ=255)`  
                                                                                    `gcTGAACCAAGAGGACCCAAGGACAGCTTGGGCGATGCTTCAAGGTATCAAGGCTGAACTAGATAAGGTCCAACCTGATGAGCAGATGACACCACAACGTGAGTGGCTAATCTCCGCACAGGAACAAGTTCAGAATCAGATGAACGCATGGACGAAAGCTCAGGCCAAGGCTC‑TGGACGATTCCATGAAGTCAATGAACAAACTTGACGTAATCAACAAGCAATTCCAGAAGCGAATCAACGGTGAGTGGGTCTCAACGGATTTTAAGGATATGCCAGTCAACGAGAACACTGGTGAGTTc                                                                                                                                                                                                                      <  1:100022/301‑1 (MQ=255)`  
                                                                                       `gAACCAAGAGGACCCAAGGACAGCTTGGGCGATGCTTCAAGGTATCAAGGCTGAACTAGATAAGGTCCAACCTGATGAGCAGATGACACCACAACGTGAGTGGCTAATCTCCGCACAGGAACAAGTTCAGAATCAGATGAACGCATGGACGAAAGCTCAGGCCAAGGCTC‑TGGACGATTCCATGAAGTCAATGAACAAACTTGACGTAATCAACAAGCAATTCCAGAAGCGAATCAACGGTGAGTGGGTCTCAACGGATTTTAAGGATATGCCAGTCAACGAGAACACTGGTGAGTTCAAg                                                                                                                                                                                                                   <  1:598158/301‑1 (MQ=255)`  
                                                                                       `gAACCAAGAGGACCCAAGGACAGCTTGGGCGATGCTTCAAGGTATCAAGGCTGAACTAGATAAGGTCCAACCTGATGAGCAGATGACACCACAACGTGAGTGGCTAATCTCCGCACAGGAACAAGTTCAGAATCAGATGAACGCATGGACGAAAGCTCAGGCCAAGGCTC‑TGGACGATTCCATGAAGTCAATGAACAAACTTGACGTAATCAACAAGCAATTCCAGAAGCGAATCAACGGTGAGTGGGTCTCAACGGATTTTAAGGATATGCCAGTCAACGAGAACACTGGTGAGTTCAAg                                                                                                                                                                                                                   >  1:641445/1‑301 (MQ=255)`  
                                                                                         `aCCAAGAGGACCCAAGGACAGCTTGGGCGATGCTTCAAGGTATCAAGGCTGAACTAGATAAGGTCCAACCTGATGAGCAGATGACACCACAACGTGAGTGGCTAATCTCCGCACAGGAACAAGTTCAGAATCAGATGAACGCATGGACGAAAGCTCAGGCCAAGGCTC‑TGGACGATTCCATGAAGTCAATGAACAAACTTGACGTAATCAACAAGCAATTCCAGAAGCGAATCAACGGTGAGTGGGTCTCAACGGATTTTAAGGATATGCCAGTCAACGAGAACACTGGTGAGTTCAAGCa                                                                                                                                                                                                                 <  1:478303/301‑1 (MQ=255)`  
                                                                                         `aCCAAGAGAACCAAAGGACAGCTTGGGCGAAGCTTCAAGGTATCAAGGCGGAACTAGATAAGGTCCAACCTGATGAGCAGATGACACCACAACGTGAGTGGCTAATCTCCGCACAGGAACAAGTTCAGAATCAGATGAACGCATGGAAGAAAGCTCAGGCCAAGGCTC‑TGGACGATTCCATGAAGTCAATGAACAAACTTGACGTAATCAACAAGCAATTCCAGAAGCTAATCAACGGTGAGTGGGTCTCAACGGATTTTAAGGATATGCCAGTCAACGATAACACTGGTGAGTTCAAGCa                                                                                                                                                                                                                 <  1:343380/301‑1 (MQ=255)`  
                                                                                                `ggACCCAAGGACAGCTTGGGCGATGCTTCAAGGTATCAAGGCTGAACTAGATAAGGTCCAACCTGATGAGCAGATGACACCACAACGTGAGTGGCTAATCTCCGCACAGGAACAAGTTCAGAATCAGATGAACGCATGGACGAAAGCTCAGGCCAAGGCTC‑TGGACGATTCCATGAAGTCAATGAACAAACTTGACGTAATCAACAAGCAATTCCAGAAGCGAATCAACGGTGAGTGGGTCTCAACGGATTTTAAGGATATGCCAGTCAACGAGAACACTGGTGAGTTCAAGCATAGCGaa                                                                                                                                                                                                          >  1:700801/1‑300 (MQ=255)`  
                                                                                                   `cccAAGGACAGCTTGGGCGATGCTTCAAGGTATCAAGGCTGAACTAGATAAGGTCCAACCTGATGAGCAGATGACACCACAACGTGAGTGGCTAATCTCCGCACAGGAACACGTTCAGAATCAGATGAACGCATGGACGAAAGCTCAGGCCAAGGCTC‑TTGACGATTCCATGAAGTCAATGAACAAACTTGACGTAATCAACAAGCAATTCCAGAAGCGAATCAACGGTGAGTGGGTCTCAACGGATTTTAAGGATATGCCAGTCAACGAGAACACTGGTgctatctcttatacacatctc                                                                                                                                                                                                       >  1:256634/1‑281 (MQ=255)`  
                                                                                                   `cccAAGGACAGCTTGGGCGATGCTTCAAGGTATCAAGGCTGAACTAGATAAGGTCCAACCTGATGAGCAGATGACACCACAACGTGAGTGGCTAATCTCCGCACAGGAACAAGTTCAGAATCAGATTAACGCATGGACGAAAGCTCAGGCCAAGGCTC‑TGGACGATTCCATGAAGTCAATGAACAAACTTGACGTAATCAACAAGCAATTCCAGAAGCGAATCAACGGTGAGTGGGTCTCAACGGATTTTAAGGATATGCCAGTCAACGAGAACACCGGTGAGTTCAAGCATAGCGATATg                                                                                                                                                                                                       >  1:359548/1‑301 (MQ=255)`  
                                                                                                   `cccAAGGACAGCTTGAGCGATGCTTCAAGGTATCAAGGCTGAACTAGATAAGGTCCAACCTGATGAGCAGATGACACCACAACGTGAGTGGCTAAGCTCCGCACAGAAACAAGTTCAGAAACAGATGAACGCAAGGACGAAAGCTCAGGCCAAGGCTC‑TGGACGATTCCATGAAGTCAATGAACAAACTTGACGTAATCAACAAGCAATTCCAGAAGCGAATCAACGGTGAGTGGGTCTCAACGGATTTTAAGGATATGCCAGTCAACGAGAACACTGGTGAGTTCAAGCATAGCGATATg                                                                                                                                                                                                       <  1:650182/301‑1 (MQ=255)`  
                                                                                                    `ccAAGGACAGCTTGGGCGATGCTTCAAGGTATCAAGGCTGAACTAGATAAGGTCCAACCTGATGAGCAGATGACACCACAACGTGAGTGGCTAATCTCCGCACAGGAACAAGTTCAGAATCAGATGAACGCATGGACGAAAGCTCAGGCCAAGGCTC‑TGGACGATTCCATGAAGTCAATGAACAAACTTGACGTAATCAACAAGCAATTCCAGAAGCGAATCAACGGTGAGTTGGTCTCAACGGATTTTAAGGATATGCCAGTCAACGAGAACACTGGTGAGTTCAAGCATAGCGATATgg                                                                                                                                                                                                      <  1:271209/301‑1 (MQ=255)`  
                                                                                                    `ccAAGGACAGCTTGGGCGATGCTTCAAGGTATCAAGGCTGAACTAGATAAGGTCCAACCTGATGAGCAGATGACACCACAACGTGAGTGGCTAATCTCCGCACAGGAACAAGTTCAGAATCAGATGAACGCATGGACGAAAGCTCAGGCCAAGGCTC‑TGGACGATTCCATGAAGTCAATGAACAAACTTGACGTAATCAACAAGCAATTCCAGAAGCGAATCAACGGTGAGTGGGTCTCAACGGATTTTAAGGATATGCCAGTCAACGAGAACACTGGTGAGTTCAAGCATAGCGATATgg                                                                                                                                                                                                      <  1:93873/301‑1 (MQ=255)`  
                                                                                                         `gtgtataagagacaggCTTCAAGGTATCAAGGCTGAACTAGATAAGGTCCAACCTGATGAGCAGATGACACCACAACGTGAGTGGCTAATCTCCGCACAGGAACAAGTTCAGAATCAGATGAACGCATGGACGAAAGCTCAGGCCAAGGCTC‑TGGACGATTCCATGAAGTCAATGAACAAACTTGACGTAATCAACAAGCAATTCCAGAAGCGAATCAACGGTGAGTGGGTCTCAACGGATTTTAAGGATATGCCAGTCAACGAGAACACTGGTGAGTTCAAGCATAGCGATATGGTTAAc                                                                                                                                                                                                 <  1:124428/286‑1 (MQ=255)`  
                                                                                                          `aCAGCTTGGGCGATGCTTCAAGGTATCAAGGCTGAACTAGATAAGGTCCAACCTGATGAGCAGATGACACCACAACGTGAGTGGCTAATCTCCGCACAGGAACAAGTTCAGAATCAGATGAACGCATGGACGAAAGCTCAGGCCAAGGCTC‑TGGACGATTCCATGAAGTCAATGAACAAACTTGACGTAATCAACAAGCAATTCCAGAAGCGAATCAACGGTGAGTGGGTCTCAACGGATTTTAAGGATATGCCAGTCAACGAGAACACTGGTGAGTTCAAGCATAGCGATATGGTTAACt                                                                                                                                                                                                <  1:278934/301‑1 (MQ=255)`  
                                                                                                                      `aTGCTTCAAGGTATCAAGGCTGAACTAGATAAGGTCCAACCTGATGAGCAGATGACACCACAACGTGAGTGGCTAATCTCCGCACAGGAACAAGTTCAGAATCAGATGAATGCATGGACGAAAGCTCAGGCCAAGGCTC‑TGGACGATTCCATGAAGTCAATGAACAAACTTGACGTAATCAACAAGCAATTCCAGAAGCGAATCAACGGTGAGTGGGTCTCAACGGATTTTAAGGATATGCCAGTCAACGAGAACACTGGTGAGTTCAAGCATAGCGATATGGTTAACTACGCCAATaaga                                                                                                                                                                                    <  1:514976/301‑1 (MQ=255)`  
                                                                                                                        `gCTTCAAGGTATCAAGGCTGAACTAGATAAGGTCCAACCTGATGAGCAGATGACACCACACCGTGAGTGGCTAATCACCGCACAGGAACAAGTTCAGAATCAGATGAACGCATGGACGAAAGCTCAGGCCAAGGCTC‑TGGACGACTCCATGAAGTCAATGAACAAACTTGACGTAATCAACAAGCAATTCCAGAAGCGAATCAACGGAGAGTGGGTCTCAACGGATTTTAAGGATATGCCAGTCAACGAGAACACTGGTGAGTTCAAGCATAGCGATATGGTTAACTACGCCAATaagaag                                                                                                                                                                                  >  1:350778/1‑301 (MQ=255)`  
                                                                                                                        `gCTTCAAGGTATCAAGGCTGAACTAGATAAGGTCCAACCTGATGAGCAGATGACACCACAACGTGAGTGGCTAATCTCCGCACAGGAACAAGTTCAGAATCAGATGAACGCATGGACGAAAGCTCAGGCCAAGGCTC‑TGGACGATTCCATGAAGTCAATGAACAAACTTGACGTAATCAACAAGCAATTCCAGAAGCGAATCAACGGTGAGTGGGTCTCAACGGATTTTAAGGATATGCCAGTCAACGAGAACACTGGTGAGTTCAAGCATAGCGATATGGTTAACTACGCCAATaagaag                                                                                                                                                                                  >  1:484780/1‑301 (MQ=255)`  
                                                                                                                        `gCTTCAAGGTATCAAGGCTGAACTAGATAAGGTCCAACATGATGAGCAGATGACACCAAAACGTGAGTGGCTAATCTCCGCACAGGAACAAGTTCAGAATCAGATGAACGCATGGACGAAAGCTCAGGCAAAGGCTC‑TGGACGATTCCATGAAGTCAATGAACAAACTTGACGTAATCAACAAGCAATTCCAGAAGCGAATCAACGGTGAGTGGGTCTCAACGGATTTTAAGGATATGCCAGTCAACGAGAACACTGGTGAGTTCAAGCATAGCGATATGGTTAACTACGCCAATaagaag                                                                                                                                                                                  <  1:552676/301‑1 (MQ=255)`  
                                                                                                                                   `gtataagagacagaGATAAGGTCCAACCTGATGAGCAGATGACACCACAACGTGAGTGGCTAATCTCCGCACAGGAACAAGTTCAGAATCAGATGAACGCATGGACGAAAGCTCAGGCCAATGCTC‑TTGACGATTCCATGAAGTCAATGAACAAACTTGACGTAATCAACAAGCAATTCCAGAAGCGAATCAACGGTGAGTGGGTCTCAACGGATTTTAAGGATATGCCAGTCAACGAGAACACTGGTGAGTTCAAGCATAGCGATATGGTTAACTACGCCAATAAGAAGCTCGCTGAGAt                                                                                                                                                                       <  1:54525/288‑1 (MQ=255)`  
                                                                                                                                   `ccAAGGCTGAACTAGATAAGTTCCCACCTGATAGGCAACTGACACCACAACGTGAGTGGCCAATCTCCGCACAGGAACAAGTTCAGAATCAGATGAACGCATGGACGAAAGCTCAGGCCAAGGCTC‑TGGACGATTCCATTAAGTCAATGAACAAACTTCACGTAATCAACAAGCAATTCCAGAAGCGAATCAACGGTGAGTGGGTCTCAACGGATTTTAAGGATATGCCAGTCAACTAGAACACTGGTGAGTTCAAGCATAGCGATATGGTTAACTACGCCAATAAGAAGCTCGCTGAGAt                                                                                                                                                                       <  1:390497/300‑1 (MQ=255)`  
                                                                                                                                     `aagacggAACTAGATAAGGTCCAACCTGATGAGCAGATTACACCACAACGTGAGTGGCTAATCGCCGCACAGGAACAAGTTCAGAATCAGGTGAACGCATGGACTAAAGCTCAGGCCAAGGCTC‑TGGACGATTCCATGGAGTCAATGAACAATCTTGACGTAATCAACAAGCAATTCCAGAAGCGAATCAACGGTGAGTGGGTCTCAACGGATTTTAAGAATATGCCAGTCAACGAGAACACTGGTGAGTTCAAGCATAGCGATATGGTTAACTACGCCAATAAGAAGCTCGCTGAGATTg                                                                                                                                                                     <  1:664240/295‑1 (MQ=255)`  
                                                                                                                                       `acaggAACTAGATAAGGTCCAACCTGATGAGCAGATGACACCACAACGTGAGTGGCTAATCTCCGCACAGGAACAAGTTCAGAATCAGATGAACGCATGGACGAAAGCTCAGGCCAAGGCTC‑TGGACGATTCCATGAAGTCAATGAACAAACTTGACGTAATCAACAAGCAATTCCAGAAGCGAATCAACGGTGAGTGGGTCTCAACGGATTTTAAGGATATGCCAGTCAACGAGAACACTGGTGAGTTCAAGCATAGCGATATGGTTAACTACGCCAATAAGAAGCTCGCTGAGATTGAc                                                                                                                                                                   <  1:131018/297‑1 (MQ=255)`  
                                                                                                                                         `gatgtgtataagagacagaaCCTGATGAGCAGATGACACCACAACGTGAGTGGCTAATCTCCGCACAGGAACAAGTTCAGAATCAGATGAACGCATGGACGAAAGCTCAGGCCAAGGCTC‑TGGACGATTCCATGAAGTCAATGAACAAACTTGACGTAATCAACAAGCAATTCCAGAAGCGAATCAACGGAGAGTGGGTCTCAACGGATTTTAAGGATATGCCAGTCAACGAGAACACTGGTGAGTTCAAGCATAGCGATATGGTTAACTACGCCAATAAGAAGCTCGCTGAGATTGACAg                                                                                                                                                                 <  1:7237/283‑1 (MQ=255)`  
                                                                                                                                         `aggAACTAGATAAGGTCCAACCTGATGAGCAGATGACACCACAACGTGAGTGGCTAATCTCCGAACAGGAACAAGATCAGAATCAGATGAACGCATGGACGAAAGCTCAGGCCAAGGCTC‑TGGACGATTCCATGAAGTCAATGAACAAACTTGACGTAATCAACAAGCAATTCCAGAAGCGAATCAACGGTGAGTGGGTCTCAACGGATTTTAAGGATATGCCAGTCAACGAGAACACTGGTGAGTTCAAGCATAGCGATATGGTTAACTACGCCAATAAGAAGCTCGCTGAGATTGACAg                                                                                                                                                                 <  1:571275/299‑1 (MQ=255)`  
                                                                                                                                           `gAACTAGATAAGGTCCAACCTGATGAGCAGATGACACCACAACGTGAGTGGCTAATCTCCGCACAGGAACAAGTTCAGAATCAGATGAACGCATGGACGAAAGCTCAGGCCAAGGCTC‑TGGACGATTCCATGAAGTCAATGAACAAACTTGACGTAATCAACAAGCAATTCCAGAAGCGAATCAACGGTGAGTGGGTCTCAACGGATTTTAAGGATATGCCAGTCAACGAGAACACTGGTGAGTTCAAGCATAGCGATATGGTTAACTACGCAAATAAGAAGCTCGCTGAGATTGACAGTa                                                                                                                                                               >  1:365774/1‑301 (MQ=255)`  
                                                                                                                                            `aaagagacagggTCCAACCTGATGAGCAGATGACACCACAACGTGAGTGGCTAATCTCCGCACAGGAACAAGTAAAGAATCAGATGAACGCATGGACGAAAGCTCAGGCCAAGGCTC‑TGGACGATTACATGAAGTCAATGAACAAACTTGACGTAAGCAACAAGCAAATCCAGAAGCGAATCAACGGTGAGTGGGACTCAACGGATTTTAAGGATATGCCAGTCAACGAGAACACTGGTGAGTTCAAGCATAGCGATATGGTTAACTACGCCAATAAGAAGCTCGCTGAGATTGACAGTAt                                                                                                                                                              <  1:500478/291‑1 (MQ=255)`  
                                                                                                                                              `cTAGATAAGGTCCAACCTGATGAGCAGATGACACCACAACGTGAGTGGCTAATATCCGCACAGGAACAAGTTCAGAATCAGATGAACGCCTGGACGAAAGCTCAGGCCAAGGCTC‑TGGACGATTCCATGAAGTCAATGAACAAACTTGACGTAATCAACAAGCAATTCCAGAAGCGAATCAACGGTGATTGGGTCTCAACGGATTTTAAGGATATGCCAGTCAACGAGAACACTGGTGAGTTCAAGCATAGCGATATGGTTAACTACGCCAATAAGAAGCTCGCTGCGCTTGACAGTATgg                                                                                                                                                            >  1:812825/1‑301 (MQ=255)`  
                                                                                                                                                `aGATAAGGTCCAACCTGATGAGCAGATGACACCACAACGTGAGTGGCTAATCTCCGCACAGGAACAAGTTCAGAATCAGATGAACGCATGGACGAAAGCTCAGGCCAAGGCTC‑TGGACGATTCCATGAAGTCAATGAACAAACTTGACGTAATCAACAAGCAATTCCAGAAGCGAATCAACGGTGAGTGGGTCTCAACGGATTTTAAGGATATGCCAGTCAACGAGAACACTGGTGAGTTCAAGCATAGCGATATGGTTAACTACGCCAATAAGAAGCTCGCTGAGATTGACAGTATGGAc                                                                                                                                                          <  1:73053/301‑1 (MQ=255)`  
                                                                                                                                                 `gATAAGGTCCAACCTGATGAGCAGATGACACCACAACGTGAGTGGCTAATCTCCGCACAGGAACAAGTTCAGAATCAGATGAACGCATGGACGAAAGCTCAGGCCAAGGCTC‑TGGACGATTCCATGAAGTCAATGAACAAACTTGACGTAATCAACAAGCAATTCCAGAAGCGAATCAACGGTGAGTGGGTCTCAACGGATTTTAAGGATATGCCAGTCAACGAGAACACTGGTGAGTTCAAGCATAGCGATATGGTTAACTACGCCAATAAGAAGCTCGCTGAGATTGACAGTATGGACa                                                                                                                                                         >  1:312777/1‑301 (MQ=255)`  
                                                                                                                                                 `gATAAGGTCCAACCTGATGAGCAGATGACACCACAACGTGAGTGGCTAATCTCCGCACAGGAACAAGTTCAGAATCAGATGAACGCATGGACGAAAGCTCAGGCCAAGGCTC‑TGGACGATTCCATGAAGTCAATGAACAAACTTGACGTAATCAACAAGCAATTCCAGAAGCGAATCAACGGTGAGTGGGTCTCAACGGATTTTAAGGATATGCCAGTCAACGAGAACACTGGTGAGTTCAAGCATAGCGATATGGTTAACTACGCCAATAAGAAGCTCGCTGAGATTGACAGTATGGACa                                                                                                                                                         >  1:584521/1‑301 (MQ=255)`  
                                                                                                                                                      `ggTCCAACCTGATGAGCAGATGACACCACAACGTGAGTGGCTAATCTCCGCACAGGAACAAGTTCAGAATCAGATGAACGCATGGACGAAAGCTCAGGCCAAGGCTC‑TGGACGATTCCATGAAGTCAATGAACAAACTTGACGTAATCAACAAGCAATTCCAGAAGCGAATCAACGGTGAGTGGGTCTCAACGGATTTTAAGGATATGCCAGTCAACGAGAACACTGGTGAGTTCAAGCATAGCGATATGGTTAACTACGCCAATAAGAAGCTCGCTGAGATTGACAGTATGGACactgtc                                                                                                                                                    >  1:784717/1‑296 (MQ=255)`  
                                                                                                                                                        `tCCAACCTGATGATCAGATGACACCACAACGTGAGTGGCTAATCTCCGCACAGGAACAAGTTCAGAATCAGATGAACGCATGGACGAAAGCTCAGGCCAAGGCTC‑TGGACGATTCCATGAAGTCAATGAACAAACTTGACGTAATCAACAAGCAATTCCAGAAGCGAATCAACGGTGAGTGGGTCTCAACGGATTTTAAGGATATGCCAGTCAACGAGAACACTGGTGAGTTCAAGCATAGCGATATGGTTAACTACGCCAATAAGAAGCTCGCTGAGATTGACAGTATGGACATTCCAGa                                                                                                                                                  <  1:180762/301‑1 (MQ=255)`  
                                                                                                                                                          `cAACCTGATGAGCAGAGGACACCACAACGTGAGTGGCTAATCTCCGCACAGGAACAAGTTCAGAATCAGATGAACGCATGGACGAAAGCTCAGGCCAAGGATC‑TGGACGATTCCATGAAGTCAATGAACAAACTTGACGTAATCAACAAGCAATTCCAGAAGCGAATCAACGGTGAGTGGGTCTCAACGGATTTTAAGGATATGCCAGTCAACGAGAACACTGGTGAGTTCAAGCATAGCGATATGGTTAACTACGCCAATAAGAAGCTCGCTGAGATTGACAGTATGGACATTCCAGACg                                                                                                                                                <  1:456837/301‑1 (MQ=255)`  
                                                                                                                                                           `aaCCTGATGAGCAGATGACACCACAACGTGAGTGGCTAATCTCCGCACAGGAACAAGTTCAGAATCAGATGAACGCATGGACGAAAGCTCAGGCCAAGGCTC‑TGGACGATTCCATGAAGTCAATGAACAAACTTGACGTAATCAACAAGCAATTCCAGAAGCGAATCAACGGTGAGTGGGTCTCAACGGATTTTAAGGATATGCCAGTCAACGAGAACACTGGTGAGTTCAAGCATAGCGATATGGTTAACTACGCCAATAAGAAGCTCGCTGAGATTGACAGTATGGACATTCAAGACgg                                                                                                                                               <  1:417376/301‑1 (MQ=255)`  
                                                                                                                                                           `aaCCTGAGGAGCAGATGACACCACAACGTGAGTGGCTAATCTCCGCACAGGAACAAGTTCAGAATCAGATGAACGCATGGACGAAAGCTCAGGCCAAGGCTC‑TGGACGATTCCATGAAGTCAATGAACAAACTTGACGTAATCAACAAGCAATTCCAGAAGCGAATCCACGGTGAGTGGGTCTCAACGGATTTTAAGGATATGCCAGTCAACGAGAACACTGGTGAGTTCAAGCATAGCGATATGGTTAACTACGCCAATAAGAAGCTCGCTGAGATTGACAGTATGGACATTCCAGACgg                                                                                                                                               <  1:827650/301‑1 (MQ=255)`  
                                                                                                                                                                `gatgaGCAGATGACACCACAACGTGAGTGGCTAATCTCCGCACAGGAACAAGTTCAGAATCAGATGAACGCATGGACGAAAGCTCAGGCCAAGGCTC‑TGGACGATTCCATGAAGTCAATGAACAAACTTGATGTAATCAACAAGCAATTCCAGCAGCGAATCAACGGTGAGTGGGTCTCAATGGATTTTAAGGATATGCCAGTCAACGAGAACACTGGTGAGTTCAAGCATAGCGATATGGTTAACTACGCCAATAAGAAGCGCGCTGAGATTGACAGAATGGCCATTCCAGACGGGGCCa                                                                                                                                          >  1:174117/1‑301 (MQ=255)`  
                                                                                                                                                                 `atgaGCAGATGACACCACAACGTGAGTGGCTAATCTCCGCACAGGAACAAGTTCAGAATCAGATGAACGCATGGACGAAAGCTCAGGCCAAGGCTC‑TGGACGATTCCATGAAGTCAATGAACAAACTTGACGTAATCAACAAGCAATTCCAGAAGCGAATCAACGGTGAGTGGGTCTCAACGGATTTTAAGGATATGCCAGTCAACGAGAACACTGGTGAGTTCAAGCATAGCGATATGGTTAACTACGCCAATAAGAAGCTCGCTGAGATTGACAGTATGGACATTCCAGACGGTGCCaa                                                                                                                                         <  1:424992/301‑1 (MQ=255)`  
                                                                                                                                                                   `gaGCAGATGACACCACAACGTGAGTGGCTAATCTCCGCACAGGAACAAGTTCAGAATCAGATGAACGCATGGACGAAAGCTCAGGCCAAGTCTC‑TGGACGATTCCATGAAGTCAATGAACAAACTTGACGTAATCAACAAGCAATTCCAGAAGCGAATCAACGGTGAGTGGGTCTCAACGGATTTTAAGGATATGCCAGTCAACGAGAACACTGGTGAGTTCAAGCATAGCGATATGGTTAACTACGCCAATAAGAAGCTCGCTGAGATTGACAGTAGGGACATTCCAGACGGTGCCAAgg                                                                                                                                       >  1:604912/1‑301 (MQ=255)`  
                                                                                                                                                                   `aaagagacagcaccacAACGTAAGTGGCTAATCACCGCACAAGAACAAGTTCAGAATCAGATGAACGAATGGACGAAAGCTCAGGCCAAGGCTC‑TGGACGATTCCATGAAGTCAATGAACAAACTTGACGTAATCAACAAGCAATTCCAGAAGCGAATCAACGGTGAGTGGGTCTCAACGGATTTTAAGGATATGCCAGTCAACGAGAACACTGGTGAGTTCAAGCATATCGATATGGTTAACTACGCCAATAAGAAGCTCGCTGAGATTGACAGTATGGACATTCCAGACGGTGCCAAgg                                                                                                                                       <  1:562823/291‑1 (MQ=255)`  
                                                                                                                                                                     `gCAGATGACACCACAACGTGAGTGGCTAATCTCCGCACAGGAACAAGTTCAGAATCAGATGAACGCATGGACGAAAGCTCAGGCCAAGGCTC‑TGGACGATTCCATGAAGTCAATGAACAAACTTGACGTAATCAACAAGCAATTCCAGAAGCGAATCAACGGTGAGTGGGTCTCAACGGATTTTAAGGATATGCCAGTCAACGAGAACACTGGTGAGTTCAAGCATAGCGATATGGTTAACTACGCCAATAAGAAGCTCGCTGAGATTGACAGTATGGACATTCCAGACGGTGCCAAGGAt                                                                                                                                     >  1:410886/1‑301 (MQ=255)`  
                                                                                                                                                                     `acAGATGACACCACAACGTGAGTGGCTAAACTCCGCACAGGAACAAGTTCAGAATCAGATGAACGCATGGACGAAAGCTCAGGCCAAGGCTC‑TGGACGATTCCATGAAGTCAATGAACAAACTTGACGTAATCAACAAGCAATTCCAGAAGCGAATCAACGGTGAGTGGGTCACAACGGATTTTAAGGATATGCCAGTCAACGAGAACACTGGTGAGTTCAAGCATAGCGATATGGTTAACTACGCCAATAAGAAGCTCGCTGAGATTGACAGTATGGACATTCCAGACGGTGCCAAGGAt                                                                                                                                     <  1:442157/300‑1 (MQ=255)`  
                                                                                                                                                                        `gATGACACCACAACGTGAGTGGCTAATCTCCGCACAGGAACAAGTTCAGAATCAGATGAACGCATGGACGAAAGCTCAGGCCAAGGCTC‑TGGACGATTCCATGAAGTCAATGAACAAACTTGACGTAATCAACAAGCAATTCCAGAAGCGAATCAACGGTGAGTGGGTCTCAACGGATTTTAAGGATATGCCAGTCAACGAGAACACTGGTGAGTTCAAGCATAGCGATATGGTTAACTACGCCAATAAGAAGCTCGCTGAGATTGACAGTATGGACATTCCAGACGGTGCCaactgtctc                                                                                                                                  >  1:685388/1‑294 (MQ=255)`  
                                                                                                                                                                        `gATGACACCACAACGTGAGTGGCTAATCTCCGCACAGGAACAAGTTCAGAATCAGATGAACGCATGGACGAAAGCTCAGGCCAAGGCTC‑TGGACGATTCCATGAAGTCAATGAACAAACTTGACGTAATCAACAAGCAATTCCAGAAGCGAATCAACGGTGAGTGGGTCTCAACGGATTTTAAGGATATGCCAGTCAACGAGAACACTGGTGAGTTCAAGCATAGCGATATGGTTAACTACGCCAATAAGAAGCTCGCTGAGATTGACAGTATGGACATTCCAGACGGTGCCAAGGATGcc                                                                                                                                  >  1:539245/1‑300 (MQ=255)`  
                                                                                                                                                                          `tGACACCACAACGTGAGTGGCTAATCTCCGCACAGGAACAAGTTCAGAATCAGATGAACGCATGGACGAAAGCTCAGGCCAAGGCTC‑TGGACGATTCCATGAAGTCAATGAACAAACTTGACGTAATCAACAAGCAATTCCAGAAGCGAATCAACGGTGAGTGGGTCTCAACGGATTTTAAGGATATGCCAGTCAACGAGAACACTGGTGAGTTCAAGCATAGCGATATGGTTAACTACGCCAATAAGAAGCTCGCTGAGATTGACAGTATGGACATTCCAGACGGTGCCAAGGCTgtctc                                                                                                                                >  1:285554/1‑297 (MQ=255)`  
                                                                                                                                                                          `ggACACCACAACGTGAGTGGCTAATCTCCGCACAGGAACAAGTTCAGAATCAGATGAACGCATGGACGAAAGCTCAGGCCAAGGCTC‑TGGACGATTCCATGAAGTCAATGAACAAACTTGACGTAATCAACAAGCAATTCCAGAAGCGAATCAACGGTGAGTGGGTCTCAACGGATTTTAAGGATATGCCAGTCAACGAGAACACTGGTGAGTTCAAGCATAGCGATATGGTTAACTACGCCAATAAGAAGCTCGCTGAGATTGACAGTATGGACATTCCAGACGGTGCCAAGGATGCTAt                                                                                                                                <  1:225654/300‑1 (MQ=255)`  
                                                                                                                                                                             `caccacAACGTGAGTGGCTAATCTCCGCACAGGAACAAGTTCAGAATCAGATGAACGCATGGACGAAAGCTCAGGCCAAGGCTC‑TGGACGATTCCATGAAGTCAATGAACAAACTTGACGTAATCAACAAGCAATTCCAGAAGCGAATCAACGGTGAGTGGGTCTCAACGGATTTTAAGGATATGCCAGTCAACGAGAACACTGGTGAGTTCAAGCATAGCGATATGGTTAACTACGCCAATAAGAAGCTCGCTGAGATTGACAGTATGGACactgtctcttatacacatctccgagcccc                                                                                                                             >  1:706692/1‑273 (MQ=255)`  
                                                                                                                                                                              `accacAACGTGAGTGGCTAATCTCCGCACAGGAACAAGTTCAGAATCAGATGAACGCATGGACGAAAGCTCAGGCCAAGGCTC‑TGGACGATTCCATGAAGTCAATGAACAAACTTGACGTAATCAACAAGCAATTCCAGAAGCGAATCAACGGTGAGTGGGTCTCAACGGATTTTAAGGATATGCCAGTCAACGATAACACTGGTGAGTTCAAGCATAGCGATATGGTTAACTACGCCAATAAGAAGCTCGCTGAGATTGACAGTATGGACATTCCAGACGGTGCCAAGGCTgtctcttat                                                                                                                            >  1:73723/1‑293 (MQ=255)`  
                                                                                                                                                                                     `agacaGTGGCTAATCTCCGCACAGGAACAAGTTCAGAATCAGATGAACGCATGGACGAAAGCTCAGGCCAAGGCTC‑TGGACGATTCCATGAAGTCAATGAACAAACTTGACGTAATCAACAAGCAATTCCAGAAGCGAATCAACGGTGAGTGGGTCTCAACGGATTTTAAGGATATGCCAGTCAACGAGAACACTGGTGAGTTCAAGCATAGCGATATGGTTAACTACGCCAATAAGAAGCTCGCTGAGATTGACAGTATGGACATTCCAGACGGTGCCAAGGATGCTAtgaagttgaagt                                                                                                                     <  1:234052/297‑1 (MQ=255)`  
                                                                                                                                                                                           `tGGCTAATCTCCGCACAGGAACAAGTTCAGAATCAGATGAACGCATGGACGAAAGCTCAGGCCAAGGCTC‑TGGACGATTCCATGAAGTCAATGAACAAACTTGACGTAATCAACAAGCAATTCCAGAAGCGAATCAACGGTGAGTGGGTCTCAACGGATTTTAAGGATATGCCAGTCAACGAGAACACTGGTGAGTTCAAGCATAGCGATATGGTTAACTACGCCAATAAGAAGCTCGCTGAGATTGACAGTATGGACATTCCAGACGGTGCCAAGGATGCTATGAAGTTGAAGTACCtgt                                                                                                               >  1:138778/1‑299 (MQ=255)`  
                                                                                                                                                                                            `ggCTAATCTCCGCACAGGAACAAGTTCAGAATCAGATGAACGCATGGACGAAAGCTCAGGCCAAGGCTC‑TGGACGATTCCATGAAGTCAATGAACAAACTTGACGTAATCAACAAGCAATTCCAGAAGCGAATCAACGGTGAGTGGGTCTCAACGGATTTTAAGGATATGCCAGTCAACGAGAACACTGGTGAGTTCAAGCATAGCGATATGGTTAACTACGCCAATAAGAAGCTCGCTGAGATTGACAGTATGGACATTCCAGACGGTGCCAAGGATGCTATGAAGTTGAAGTACCTTCa                                                                                                              >  1:819413/1‑301 (MQ=255)`  
                                                                                                                                                                                             `gCTAATCTCCGCACAGGAACAAGTTCAGAATCAGATGAACGCATGGACGAAAGCTCAGGCCAAGGCTC‑TGGACGATTCTATGAAGTCAATGAACAAACTTGACGTAATCAACAAGCAATTCCAGAAGCGAATCAACGGTGAGTGGGTCTCAACGGATTTTAAGGATATGCCAGTCAACGAGAACACTGGTGAGTTCAAGCATAGCGATATGGTTAACTACGACAATAAGAAGCTCGCTGAGATTGACAGTATGGACATTCCAGACGGTGCCAAGGATGCTAtgaagtctgtctcttataca                                                                                                             >  1:10711/1‑287 (MQ=255)`  
                                                                                                                                                                                              `cTAATCTCCGCACAGGAACAAGTTCAGAATCAGATGAACGCATGGACGAAAGCGCAGGCCAAGGCTC‑TGGACGATTCCATGAAGTCAATGAACAAACTTGACGTAATCAACAAGCAATTCCAGAAGCGAATCAACGGTGAGTGGGTCTCAACGGATTTTAAGGATATGCCAGTCAACGAGAACACTGGTGAGTTCAAGCATAGCGATATGGTTAACTACGCCAATAAGAAGCTCGCTGAGATTGACAGTATGGACATTCCAGACGGTGCCAAGGATGCTATGAAGTTGAAGTACCTTCAAg                                                                                                            <  1:88145/301‑1 (MQ=255)`  
                                                                                                                                                                                                 `aTCTCCGCACAGGAACAAGTTCAGAATCAGATGAACGCATGGACGAAAGCTCAGGCCAAGGCTA‑TGGACGATTCCATGAAGTCAATGAACAAACTTGACGTAATCAACAAGCAATTCCAGAAGCGAATCAACGGTGAGTGGGTCTCAACGGATTTTAAGGATATGCCAGTCAACGAGAACACTGGTGAGTTCAAGCATAGCGATATGGTTAACTACGCCAATAAGAAGCTCGCTGAGATTGACAGTATGGACATTCCAGACGGTGCCAAGGATGCTATGAAGTTGAAGTACCTTCAAGCgg                                                                                                         >  1:116682/1‑301 (MQ=255)`  
                                                                                                                                                                                                  `tctcCGCACAGGAACAAGTTCAGAATCAGATGAACGCATGGACGAAAGCTCAGGCCAAGGCTC‑TGGACGATTCCATGAAGTCAATGAACAAACTTGACGTAATCAACAAGCAATTCCAGAAGCGAATCAACGGTGAGTGGGTCTCAACGGATTTTAAGGATATGCCAGTCAACGAGAACACTGGTGAGTTCAAGCATATCGATATGGTTAACTACGCCAATAAGAAGCTCGCTGAGATTGACAGTATGGACATTCCAGACGGTGCCAAGGATGCTATGAAGTTGAAGTACCTTCAAGCGGa                                                                                                        >  1:693692/1‑301 (MQ=255)`  
                                                                                                                                                                                                   `ctcCGCACAGGAACAAGTTCAGAATCAGATGAACGCATGGACGAAAGCTCAGGCCAAGGCTC‑TGGACGATTCCATGAAGTCAATGAACAAACTTGACGTAATCAACAAGCAATTCCAGAAGCGAATCAACGGTGAGTGGGTCTCAACGGATTTTAAGGATATGCCAGTCAACGAGAACACTGGTGAGTTCAAGCATAGCGATATGGTTAACTACGCCAATAAGAAGCTCGCTGAGATTGACAGTATGGACATTCCAGACGGTGCCAAGGATGCTATGAAGTTGAAGTACCTTCAAGCGGAc                                                                                                       >  1:15623/1‑301 (MQ=255)`  
                                                                                                                                                                                                        `aaaaGGAACAAGTTCAGAATCAGATGAACGCATGGACGAAAGTTCAGGCCAAGGCTC‑TGGACGATTCCATGAAGTCAATGAACAAACTTGACGTAATCAACAAGAAATTCCAGAAGCGAAGCAACGGTGAGTGGGTCGCAAAGGATTGTAAGGATAGGCCAGTCAACGAGAACACTGGAGAGTTCAAGCATAGCGATATGGTTAACTACGCCAATAAGAAGCTCGCTGAGATTGACAGTATGGACATTCCAGACGGTGCCAAGGATGATATGAAGTTGAAGTACCTTCAAGCGGACTCTaa                                                                                                  <  1:44858/298‑1 (MQ=255)`  
                                                                                                                                                                                                         `acaGGAACAAATTCAGAAACCAATGAACGCATGGACGAAAAGTCAGGGAAAGGGTCTTGGAC‑AATCCATGGAGTCAAAGAACAAACTTTACGTAAACAACAAGCAATTCCAAAAACCAATCAACGGTGAGTGGGTCTCAACCGATTTTAAGGATATGCCAGTCAACGGGGACACTGGTGAGTTCAAGCATTGGGATATTGTTAACTACGCCAAAAAGAAGCTCGCTGAGATTGACAGTATGGACATTCCAGACGGTGCCAAGGATGCTATGAAGTTGAAGTACCTTCAAGCGGACTCTAAg                                                                                                 <  1:217545/301‑1 (MQ=255)`  
                                                                                                                                                                                                          `caGGAACAAGTTCAGAATCAGATGAACGCATGGACGAAAGCTCAGGCCAAGGCTC‑TGGACGATTCCATGAAGTCAATGAACAAACTTGACGTAATCAACAAGCAATTCCAGAAGCGAATCAACGGTGAGTGGGTCTCAACGGATTTTAAGGATATGCCAGTCAACGAGAACACTGGTGAGTTCAAGCATAGCGATATGG‑TAACTACGCCAATAAGAAGCTCGCTGAGATTGACAGTATGGACATTCCAGACGGTGCCAAGGATGCTATGAAGTTGAAGTACCTTCAAGCGGACTCTAAGGa                                                                                               <  1:507651/301‑1 (MQ=255)`  
                                                                                                                                                                                                             `gAACAAGTTCAGAATCAGATGAACGCATGGACGAAAGCTCAGGCCAAGGCTC‑TGGACGATTCCATGAAGTCACTGAACAAACTTGACGTAATCAACAAGCAATTCCAGAAGCGAATAAACGGTGCGTGGATCTCAACGGATTATAAGAATATGCCAGTCAACGAGAACACTGGTGAGTTCAAGCATAGCGATATGGTTAACTACGCCAATAAGAAGCTCGCTGAGATTGCCAGTATGGACATTCCAGACGGTGCCAAGGATGCTATGAAGTTGAAGTACCTTCAAGCGGACTCTAAGGAct                                                                                             >  1:679276/1‑300 (MQ=255)`  
                                                                                                                                                                                                                        `agacaggATGAACGCATGGACGAAAGCTCAGGCCAAGGCTC‑TGGACGATTCCATGAAGTCAATGAACAAACTTGACGTAATCAACAAGCAATTCCAGAAGCGAATCAACGGTGAGTGGGTCTCAACGGATTTTAAGGATATGCCAGTCAACGAGAACACTGGTGAGTTCAAGCATAGCGATATGGTTAACTACGCCAATAAGAAGCTCGCTGAGATTGACAGTATGGACATTCCAGATGGTGCCAAGGATGCTATGAAGTTGAAGTACCTTCAAGCGGACTCTAAGGACGGAGCATTCCGt                                                                                  <  1:262658/295‑1 (MQ=255)`  
                                                                                                                                                                                                                          `aTCAGATGAACGCATGGACGAAAGCTCAGGCCAAGGCTC‑TGGACGATTCCATGAAGTCAATGAACAAACTTGACGTAATCAACAAGCAATTCCAGAAGCGAATCAACGGTGAGTGGGTCTCAACGGATTTTAAGGATATGCCAGTCAACGAGAACACTGGTGAGTTCAAGCATAGCGATATGGTTAACTACGCCAATAAGAAGCTCGCTGAGATTGACAGTATGGACATTCCAGACGGTGCCAAGGATGCTATGAAGTTGAAGTACCTTCAAGCGGACTCTAAGGACGGAGCATTCCGTAc                                                                                <  1:307164/301‑1 (MQ=255)`  
                                                                                                                                                                                                                             `aGATGAACGCATGGACGAAAGCTCAGGCCAAGGCTC‑TGGACGATTCCATGAAGTCAATGAACAAACTTGACGTAATCAACAAGCAATTCCAGAAGCGAATCAACGGTGAGTGGGTCTCAACGGATTTTAAGGATATGCCAGTCAACGAGAACACTGGTGAGTTCAAGCATAGCGATATGGTTAACTACGCCAATAAGAAGCTCGCTGAGATTGACAGTATGGACATTCCAGACGGTGCCAAGGATGCTATGAAGTTGAAGTACCTTCAAGCGGACTCTAAGGACGGAGCATTCCGTACAGc                                                                             <  1:210437/301‑1 (MQ=255)`  
                                                                                                                                                                                                                                        `tGGACGAAAGCTCAGGCCAAGGCTC‑TGGACGATTCCATGAAGTCAATGAACAAACTTGACGTAATCAACAAGCAATTCCAGAAGCGAATCAACGGTGAGTGGGTCTCAACGGATTTTAAGGATATGCCATTCAACGAGAACACTGGTGAGTTCAAGCATAGCGATATGGTTAACTACGCCAATAAGAAGCTCGCTGAGATTGACAGTATGGACATTCCAGACGGTGCCAAGGATGCTATGAAGTTGAAGTACCTTCAAGCGGACTCTAAGGActgtctcttatacacatctccgagcccac                                                                  >  1:768676/1‑273 (MQ=255)`  
                                                                                                                                                                                                                                                     `aGGCCAAGGCTC‑TGGACGATTCCATGAAGTCAATGAACAAACTTGACGTAATCAACAAGCAATTCCAGAAGCGAATCAACGGTGAGTGGGTCTCAACGGATTTTAAGGATATGCCAGTCAACGAGAACACTGGTGAGTTCAAGCATAGCGATATGGTTAACTACGCCAATAAGAAGCTCGCTGAGATTGACAGTATGGACATTCCAGACGGTGCCAAGGATGCTATGAAGTTGAAGTACCTTCAAGCGGACTCTAAGGACGGAGCATTCCGTACAGCCATCGGAACCATGGTCACTGACGc                                                     <  1:299710/301‑1 (MQ=255)`  
                                                                                                                                                                                                                                                     `aGGCAAAGGCTC‑TGGACGATTCCATGAAGTCAATGAACAAACTTGACGTAATCAACAAGCAATTCCAGAAGCGAATCAACGGTGAGGGGGTCTCAACGGATTTTAAGGATATGCCAGTCAACGAGAACACTGGTGAGTTCAAGCATAGCGATATGGTTAACTACGCCAATAAGAAGCTCGCTGAGATTGACAGTATGGACATTCCAGACGGTGCCAAGGATGCTATGAAGTTGAAGTACCTTCAAGCGGACTCTAAGGACGGAGCATTCCGTACAGCCATCGGAACCATGGTCACTGACGc                                                     <  1:633247/301‑1 (MQ=255)`  
                                                                                                                                                                                                                                                      `ggCCAAGGCTC‑TGGACGATTCCATGAAGTCAATGAACAAACTTGACGTAATCAACAAGCAATTCCAGAAGCGAATCAACGGTGAGTGGGTCTCAACGGATTTTAAGGATATGCCAGTCAACGAGAACACTGGTGAGTTCAAGCATAGCGATATGGTTAACTACGCCAATAAGAAGCTCGCTGAGATTGACAGTATGGACATTCCAGACGGTGCCAAGGATGCTATGAAGTTGAAGTACCTTCAAGCGGACTCTAAGGACGGAGCATTCCGTACAGCCATCGGAACCactgtctcttataca                                                    >  1:625607/1‑287 (MQ=255)`  
                                                                                                                                                                                                                                                      `ggCCAAGGCTC‑TGGACGATTCCATGAAGTCAATGAACAAACTTGACGTAATCAACAAGCAATTCCAGAA‑CGAATCAACGGTGAGTGGGTCTCAACGGATTTTAAGGATATGCCAGTCAACGAGAACACTGGTGAGTTCAAGCATAGCGATATGGTTAACTACGCCAATAAGAAGCTCGCTGAGATTGACAGTATGGACATTCCAGACGGTGCCAAGGATGCTATGACGTTGAAGTACCTTCAAGCGGACTCTAAGGACGGAGCATTCCGTACAGCCATCGGAAcctgtctcttatacacat                                                   >  1:195786/1‑285 (MQ=255)`  
                                                                                                                                                                                                                                                             `gCTC‑TGGACGATTCCATGAAGTCAATGAACAAACTTGACGTAATCAACAAGCAATTCCAGAAGCGAATCAACGGTGAGTGGGTCTCAACGGATTTTAAGGATATGCCAGTCAACGAGAACACTGGTGAGTTCAAGCATAGCGATATGGTTAACTACGCCAATAAGAAGCTCGCTGAGATTGACAGTATGGACATTCCAGACGGTGCCAAGGATGCTATGAAGTTGAAGTACCTTCAAGCGGACTCTAAGGACGGAGCATTCCGTACAGCCATCGGAACCATGGTCACTGACGCTGGTCAag                                             >  1:188952/1‑301 (MQ=255)`  
                                                                                                                                                                                                                                                             `gCTC‑TGGACGATTCCATGAAGTCAATGAACAAACTTGACGTAATCAACAAGCAATTCAAGAAGCGAATCAACGGTGAGTGGGTCTCAACGGATTTTAAGGATATGCCAGTCAACGAGAACACTGGTGAGTTCAAGCATAGCGATATGGTTAACTACGCCAATAAGAAGCGCGCTGAGATTGACAGTATGGACATTCCAGACAGTGCCAAGGATGCTATGAAGATGAAGTACCTTCAAGCGGACGCTAAGGACGGAGAATTCCGTACAGCCATCGGAACCAGGGTGACTGAAGCTGGTCAag                                             >  1:470404/1‑301 (MQ=255)`  
                                                                                                                                                                                                                                                                     `aagATTCCATGAAGTCAATGAACAAACTTGACGTAATCAACAAGCAATTCCAGAAGCGAATCAACGGTGAGTGGGTCTCAACGGATTTTAAGGATATGCCAGTAAACGAGAACACTGGTGAGTTCAAGCATAGCGATATGGTTAACTACGCCAATAAGAAGCTCGCTGAGATTGACAGTATGGACATTCCAGACGGTGCCAAGGATGCTATGAAGTTGAAGTACCTTCAAGCGGACTCTAAGGACGGAGCATTCCGTACAGCCATCGGAACCATGGTCACTGACGCTGGTCAAGAGTGGTc                                      <  1:396129/299‑1 (MQ=255)`  
                                                                                                                                                                                                                                                                     `aCGATTCCATGAAGTCAATGAACAAACTTGACGTAATCAACAAGCAATTCCAGAAGCGAATCAACGGTGAGTGGGTCTCAACGGATTTTAAGGATATGCCAGTCAACGAGAACACTGGTGAGTTCAAGCATAGCGATATGGTTAACTACGCCAATAAGAAGCTTGCTGAGATTGACAGTATGGACATTCCAGACGGTGCCAAGGATGCTATGAAGTTGAAGTACCTTCAAGCGGACTCTAAGGACGGAGCATTCCGTACAGCCATCGGAACCATGGTCACTGACGCTGGTCAAGAGTGGTc                                      <  1:334923/301‑1 (MQ=255)`  
                                                                                                                                                                                                                                                                           `ccATGAAGTCAATGAACAAACTTGACGTAATCAACAAGCAATTCCAGAAGCGAATCAACGGTGAGTGGGTCTCAACGGATTTTAAGGATATGCCAGTCAACGAGAACACTGGTGAGTTCAAGCATAGCGATATGGTTAACTACGCCAATAAGAAGCTCGCTGAGATTGACAGTATGGACATTCCAGACGGTGCCAAGGATGCTATGAAGTTGAAGTACCTTCAAGCGGACTCTAAGGACGGAGCATTCCGTACAGCCATCGGAACCATGGTCACTGACGCTGGTCAAGAGTGGTCTGcctg                                <  1:577368/301‑3 (MQ=255)`  
                                                                                                                                                                                                                                                                                `aaGTCAATGAACAAACTTGACGGAAACAACAAGCAATTCCAGAAGCGAATCAACAGTGAGTGGATCTCAACGGATTTTAAGGATATGCCAGTAAACGAGAACACTGGTGATTTCAAGCATAGCGATATGGTGAACTACGCCAATAAGAAGCGCGCTGAGATTGACAGTATGGACATTACAGACGGTGCCAAGGATGCTATGAAGTTGAAGTACCTTCAAGCGGACTCTAAGGACGGAGCATTCCGTACAGCCATCGTAACCATGGTCACTGACGCTGGTCAAGAGTGGTCTGCCGATGTGa                           <  1:402692/301‑1 (MQ=255)`  
                                                                                                                                                                                                                                                                                  `gTCAATGAACAGACTTGACGTAATCAACAAGCAATTCCAGAAGCGAATCAACGGTGAGTGGGTCTCAACGGATTTTAAGGATATGCCAGTCAACGAGAACACTGGTGAGTTCAAGCATAGCGATATGGTTAACTACGCCAATAAGAAGCTAGCTGAGATTGACAGTATGGACATTCCAGACGGTGCCAAGGATGCTATGAAGTTGAAGTACCTTCAAGCGGACTCTAAGGACGGAGCATTCCGTACAGCCATCGGAACCATGGTCACTGACGCTGGTCAAGAGTGGTCTGCCGCTGTGAtt                         <  1:320415/301‑1 (MQ=255)`  
                                                                                                                                                                                                                                                                                    `cAATTAACAAACTTGACGTAATCAACAAGCAATTCCAGAAGCGAATCAACGGTGAGTGGGTCTCAACGGATTTTAAGGATATGCCAGTCAACGAGAACACTGGTGAGTTCAAGCATAGCGATATGGTTAACTACGCCAATAAGAAGCTCGCTGAGATTGACAGTATGGACATTCCAGACGGTGCCAAGGATGCTATGAAGTTGAAGTACCTTCAAGCGGACTCTAAGGACGGAGCATTCCGTACAGCCATCGGAACCATGGTCACTGACGCTGGTCAAGAGTGGTCTGTCGCTGTGATTaa                       >  1:618126/1‑301 (MQ=255)`  
                                                                                                                                                                                                                                                                                     `aaTGAACAAACTTGACGTAATCAACAAGCAATTCCAGAAGCGAATCAACGGTGAGTAGGTCTCAACGGATTTTAAGGATATGCCAGTCAACGAGAACACTGGTGAGTTCAAGCATAGCGATATGGTTAACTACGCCAATAAGAAGCTCGCTGAGATTGACAGTATGGACATTCCAGACGGTGCCAAGGATGCTATGAAGTTGAAGTACCTTCAAGCGGACTCTAAGGACGGAGCATTCCGTACAGCCATCGGAACCATGGTCACTGACGCTGGTCAAGAGTGGTCTGCCGCTGTGATTAAc                      <  1:760165/301‑1 (MQ=255)`  
                                                                                                                                                                                                                                                                                             `aaCTTGACGTAATCAACAAGCAATTCCAGAAGCGAATCAACGGTGAGTGGGTCTCAACGGATTTTAAGGATATGCCAGTCAACGAGAACACTGGTGAGTTCAAGCATAGCGATATGGTTAACTACGCCAATAAGAAGCTCGCTGAGATTGACAGTATGGACATTCCAGACGGTGCCAAGGATGCTATGAAGTTGAAGTACCTTCAAGCGGACTCTAAGGACGGAGCATTCCGTACAGCCATCGGAACCATGGTCACTGACGCTGGTCAAGAGTGGTCTGCCGCTGTGATTAACGGTAAGtt              >  1:657799/1‑301 (MQ=255)`  
                                                                                                                                                                                                                                                                                                    `cGTAATCAACAAGCAATTCCAGAAGCGAATCAACGGTGAGTGGGTCTCAACGGATTTTAAGGATATGCCAGTCAACGAGAACACTGGTGAGTTCAAGCATAGCGATATGGTTAACTACGCCAATAAGAAGCTCGCTGAGATTGACAGTATGGACATTCTAGACGGTGCCAATGATGCTATGAAGTTGAAGTACCTTCAAGCGGACTCTAAGGACGGAGCATTCCGTACAGCCATCGGAACCATGGTCACTGACGCTGGTCAAGAGTGGTCTGCCGCTGTGATTAACGGTAAGTTACCAgaa       <  1:248111/301‑1 (MQ=255)`  
                                                                                                                                                                                                                                                                                                    `cGTAATCAACAAGCAAATCCAGAAGCGAATCAACGGTGAGTGGGTCTCAACGGATTTTAAGGATATGCCAGTCAACGAGAACACTGGTGAGTTCAAGCATAGCGATATGGTTAACTACGCCAATAAGAAGCTCGCTGAGATTGACAGTATGGACATTCCAGACGGTGCCAAGGATGCTATGAAGTTGAAGTACCTTCAAGCGGACTCTAAGGACGGAGCATTCCGTACAGCCATCGGAACCATGGTCACTGACGCTGGTCAAGAGTGGTCTGCCGCTGTGATTAACGGTAAGTTACCAgaa       <  1:714932/301‑1 (MQ=255)`  

                                                                                                                                                                                                                                                                                                           `|`                                                                                                                                                                                                                                                                                                            
`CGCTCTCATGGTCACAGCACAACGTTCTCAGTTTGAGACTGACGCGAAGCTGAACGAGCAGTATCGCTTGAAGATTAACTCTGCGCTGAACCAAGAGGACCCAAGGACAGCTTGGGAGATGCTTCAAGGTATCAAGGCTGAACTAGATAAGGTCCAACCTGATGAGCAGATGACACCACAACGTGAGTGGCTAATCTCCGCACAGGAACAAGTTCAGAATCAGATGAACGCATGGACGAAAGCTCAGGCCAAGGCTC‑TGGACGATTCCATGAAGTCAATGAACAAACTTGACGTAATCGACAAGCAATTCCAGAAGCGAATCAACGGTGAGTGGGTCTCAACGGATTTTAAGGATATGCCAGTCAACGAGAACACTGGTGAGTTCAAGCATAGCGATATGGTTAACTACGCCAATAAGAAGCTCGCTGAGATTGACAGTATGGACATTCCAGACGGTGCCAAGGATGCTATGAAGTTGAAGTACCTTCAAGCGGACTCTAAGGACGGAGCATTCCGTACAGCCATCGGAACCATGGTCACTGACGCTGGTCAAGAGTGGTCTGCCGCTGTGATTAACGGTAAGTTACCAGAACGAAC  >  NC_001604.1/29188‑29784`

  

Alignment Legend

Aligned base mismatch/match (shaded by quality score): `ATCG/ATCG` `< 3 ≤` `ATCG/ATCG` `< 11 ≤` `ATCG/ATCG` `< 24 ≤` `ATCG/ATCG` `< 36 ≤` `ATCG/ATCG` `< 38 ≤` `ATCG/ATCG`

Unaligned base: `atcg`    Masked matching base: `atcg`    Alignment gap: `‑`    Deleted base: `‑`
